# Book Review

**Published:** 1886-06

**Authors:** 


We commend to the notice of our readers three handy little volumes:—Tirard's *Prescriber's Pharmacopœia*, published by J. and A. Churchill; Lewis's *Medical Vocabulary*, and Aitchison's *Synopsis of Therapeutics*, published by Pentland of Edinburgh.

---

### NEW EDITIONS.

(1) *Elements of Practical Medicine.* By A. H. CARTER. 4th Ed. Pp. 443. London: Lewis.

(2) *A Guide to Therapeutics.* By R. FARQUHARSON. 4th Ed. Pp. 394. London: Smith, Elder & Co.

(3) *Alpine Winter.* By A. TUCKER WISE. 2nd Ed. Pp. 121. London: J. & A. Churchill.

(4) *How to Use a Galvanic Battery.* By HERBERT TIBBITS. 3rd Ed. London: J. & A. Churchill.

(5) *A Guide to the New Pharmacopœia.* By PROSSER JAMES. 2nd Ed. London: J. & A. Churchill.

(6) *Diseases of the Lungs.* By Dr. POWELL. 3rd Ed. London: J. & A. Churchill.

(1) An author is quite justified in assuming that his work, having passed through four editions in five years, "meets a distinct want," as Dr. CARTER expresses himself in the preface to his book. It is surprising how much material may be put into so small space if verbosity is eschewed, repetition avoided, and unverified hypotheses ignored. We have here only absolute fact, rigidly classified and told in simple clear language. It admirably fulfils the purpose with which it was written, "to provide the student with a general introduction to the study of Medicine."

(2) It is creditable to Dr. FARQUHARSON that, in the midst of his Parliamentary labours, he has been able, so soon after the publication of the new *Pharmacopœia*, to bring his work on *Therapeutics* abreast of it. The changes in the work, the general character of which we have already described and praised, are limited almost entirely to those rendered necessary by the action of the Pharmacopœia Committee. He considers that the Committee have not discharged their duties well: what Committee ever did?

(3) It is no easy matter to hide what our Yankee friends would call a "boom" under the pretence of a scientific treatise on the medical aspects of *Alpine Winter*. The chief end of this work is to describe the advantages of the Maloja as a health resort in general, and of the Hôtel Kursaal as a place to stay at in particular. Dr. WISE's work has less of the character of an advertisement than such productions usually have. The Hôtel Kursaal seems to be absolutely the best building of its sort in existence: ventilated, warmed, and altogether set up in the latest and best lights of science, and apparently without regard to expense. The Maloja plateau has evidently a future before it. Dr. WISE's general remarks on the selection, clothing, and management of patients are short, practical, and to the point.

(4) How many books are written for the "busy practitioner!" What proportion of practitioners are busy, and how many of the busy ones read the books written for them, is not very clear. Whatever of the time which courtesy describes as being "valuable" can be spared by the practitioner to the study of electro-therapeutics, may be most profitably spent in a perusal of Dr. TIBBITS' little work. It provides, within very small compass, a well-told and thoroughly practical account of the whole art of galvanism as applied to Medicine.

(5) Dr. PROSSER JAMES' useful *Guide to the New Pharmacopœia* having been at once bought up, he now issues a second edition, printed on better paper, more strongly bound, and supplied with a useful index.

(6) Dr. POWELL's work on the *Lungs* is, as compared with the original edition published in 1878, a new production. New chapters have been added, much has been re-written, and the whole book has been recast and considerably increased in size. It is a good, sound, sensible book, dealing in an earnest, simple, and straightforward manner with a highly important class of diseases. The author wisely subordinates the theoretical to the practical, and the speculative to the assured. While we cannot complain that he overlooks modern views as to germs and their influences, we must fully admit that he does ample justice to old and tried plans of treatment. The book is well worthy of a permanent place on the shelves of even a small medical library.

